# A Prolonged Artificial Nighttime-light Dataset of China (1984-2020)

**DOI:** 10.1038/s41597-024-03223-1

**Published:** 2024-04-22

**Authors:** Lixian Zhang, Zhehao Ren, Bin Chen, Peng Gong, Bing Xu, Haohuan Fu

**Affiliations:** 1 High Performance Computing Department, National Supercomputing Center in Shenzhen, Shenzhen, China; 2 Department of Earth System Science, Tsinghua University, Beijing, 100084 China; 3 Ministry of Education Ecological Field Station for East Asian Migratory Birds, Department of Earth System Science, Institute for Global Change Studies, Tsinghua University, Beijing, 100084 China; 4 International Research Center of Big Data for Sustainable Development Goals, Beijing, 100094 China; 5 Future Urbanity & Sustainable Environment (FUSE) Lab, Division of Landscape Architecture, Faculty of Architecture, The University of Hong Kong, Hong Kong Special Administrative Region, China; 6 Urban Systems Institute, The University of Hong Kong, Hong Kong Special Administrative Region, China; 7 HKU Musketeers Foundation Institute of Data Science, The University of Hong Kong, Hong Kong Special Administrative Region, China; 8 Department of Geography, Department of Earth Sciences and Institute for Climate and Carbon Neutrality, The University of Hong Kong, Hong Kong, China; 9 Tsinghua University (Department of Earth System Science)-Xi’an Institute of Surveying and Mapping Joint Research Center for Next-Generation Smart Mapping, Beijing, 100084 China; 10 Tsinghua Shenzhen International Graduate School, Tsinghua University, Shenzhen, China

**Keywords:** Environmental economics, Environmental economics, Sustainability

## Abstract

Nighttime light remote sensing has been an increasingly important proxy for human activities. Despite an urgent need for long-term products and pilot explorations in synthesizing them, the publicly available long-term products are limited. A Night-Time Light convolutional LSTM network is proposed and applied the network to produce a 1-km annual Prolonged Artificial Nighttime-light DAtaset of China (PANDA-China) from 1984 to 2020. Assessments between modeled and original images show that on average the RMSE reaches 0.73, the coefficient of determination (R^2^) reaches 0.95, and the linear slope is 0.99 at the pixel level, indicating a high confidence in the quality of generated data products. Quantitative and visual comparisons witness PANDA-China’s superiority against other NTL datasets in its significantly longer NTL dynamics, higher temporal consistency, and better correlations with socioeconomics (built-up areas, gross domestic product, population) characterizing the most relevant indicator in different development phases. The PANDA-China product provides an unprecedented opportunity to trace nighttime light dynamics in the past four decades.

## Background & Summary

Spaceborne sensors with nighttime light (NTL) capabilities have served as an effective measure of various human activities over the past years^1–3^. In recent years, the NTL data has provided a unique perspective on the intensity of lighting, which is related to the dynamics of socioeconomic activities and urban development. The availability of long-term NTL data has triggered extensive efforts in multiple long-term research frontiers^1–3^. For instance, mapping long-term urbanization processes benefits from the unique advantage of the NTL observations spanning a relatively long period, including urban extent^4,5^, urban boundary^6,7^, impervious surface areas^8,9^, urban land use^8,10,11^, and built-up infrastructure^12–14^. Furthermore, long-term NTL datasets have proved to successfully estimate the population^15,16^, the gross domestic product (GDP)^17^ and income^18–20^, but also the poverty^21–23^ and freight traffic^24^.

NTL datasets supporting the application above mainly derive from two groups. The first group is a primary NTL data source from the Defense Meteorological Satellite Program - Operational Linescan System (DMSP-OLS), which provides valuable records of global nightscape from 1992 to 2013. It has been widely used in socioeconomic fields even though suffering from the brightness saturation in urban centers^1,25^ and the blooming effect near the urban-rural transitions^26,27^ regarding its relatively long-term historical records. However, it is no longer available after 2013, which defines its time period as permanent 1992 ~ 2013^2,28–31^. The second group of NTL dataset derived from satellites on track mostly started working since then, including Suomi National Polar-Orbiting Partnership-Visible Infrared Imaging Radiometer Suite (NPP-VIIRS), Luojia 1-01 satellite^32^, and Jilin1-03b (Jilin-1) satellite^33^ and SDGSAT^34^. As new generations of global NTL composites, they provided higher spatial resolution and fewer over-glow effects of the recorded radiance of NTL data compared to that of DMSP^28,35,36^. However, their time spans are only available since 2012 at the earliest, resulting in a relatively short period for mapping the dynamics of human activities^8,9,27,37,38^.

In all, even usable satellite NTL data has been publicly available since 1992, there is, unfortunately, no such dataset with high temporal consistency that spans from 1992 till now. The quality^39^ and the available time span^3^ of existing NTL datasets limited their capability to reflect long-term spatiotemporal dynamics of human behaviour. Per these shortcomings and urgent needs, several attempts have been made to synthesize consistent nightlight time series across different platforms and sensors, which can be classified into a new third group. Li *et al*.^37^ proposed an inter-calibration model to simulate DMSP/OLS composites from the VIIRS day-and-night band (DNB) composites by using a power function for radiometric degradation and a Gaussian low pass filter for spatial degradation (RMSE:5.00, R^2^:0.92). Zhao *et al*.^40^ conducted a sigmoid function model for generating a temporally consistent NTL dataset from 1992 to 2018 in Southeast Asia (R^2^: 0.91 in 2012, 0.94 in 2013). Li *et al*.^3^ generated an integrated and consistent NTL dataset using a sigmoid function at the global scale (1992-2018). Despite similar pilot efforts^39,41,42^, it still lacks comprehensive and systematic evaluation frameworks for assessing the quality and reliability of the generated NTL dataset. Although statistical errors have been calculated, the temporal consistency of these datasets has been seldom checked and assumed in good accordance by default, which is not the case. Neither DMSP-OLS nor NPP-VIIRS has high temporal accordance owing to its manual-like pre-process recorded in official documents^43^, let alone that of a synthesized dataset deriving from these satellite datasets with different passing times.

To produce a longer-period NTL dataset as well as develop higher temporal consistency, we recommend the potential of historical records of DMSP be fully explored, with the help of newly adopted deep learning methods followed with a temporal consistency correction model. The recent rapid development of deep learning approaches^44–46^ has provided a targeted and promising method in modeling the dependencies between the spatiotemporal dynamics of the DMSP-OLS. The LSTM architecture hereby has proved its capability in several spatiotemporally dependent applications^47–49^, which is promisingly helpful in modeling the spatial and temporal dependencies of NTL data.

Considering the abilities of existing deep learning approaches in capturing the long-range spatial and temporal dependencies of NTL data remain to be improved^50,51^, in this study, we propose a space- and time-aware approach named nighttime light convolutional long short-term memory network (NTLSTM) for modeling the relationship between dynamic changes of the long-term DMSP data followed with a temporal consistency correction method adapted from Robust LOcally WEighted Scatterplot Smoothing (RLOWESS) (Cleveland 1979). With the newly proposed method, we achieve the time series of NTL data in China spanning 1984 to 2020 for the first time, affirm its temporal consistency, name it a prolonged artificial nighttime-light dataset of China (PANDA-China), and analyze the spatiotemporal urbanization process at both national and regional scales using PANDA-China.

## Methods

### Study area and used data

In this work, we focus on China as the study area, which has experienced different levels of fast urban development in different regions over the past four decades. The relatively different levels of development in China are suitable for assessing both the proposed method as well as the newly generated PANDA-China.

DMSP-OLS time-series data from 1992 to 2012 is retrieved from the National Geophysical Data Center (NGDC) at the National Oceanic and Atmospheric Administration (NOAA) website (https://www.ncei.noaa.gov/products/dmsp-operational-linescan-system). In brief, DMSP-OLS sensors have a unique capability to detect visible lights from country-sides, towns, cities, and other sites with persistent lighting and exclude the effect of accidental noise such as stray light, lightning, lunar illumination, and cloud cover. Their digital number (DN) values range from 0 to 63. Before the experiments, the temporal consistency has been improved through ridgeline regression, and DMSP-2013 is excluded considering its quality^50,51^.

As for the training and evaluation period of deep learning, the training and evaluation material is generated by randomly cropping the raw DMSP NTL images into patches with the size of 1,024 × 1,024 pixels. The generated patches are divided into training, validating, and testing materials in a proportion of 7:2:1.

Nine ancillary data sets are collected to help validate the accuracy or performance of PANDA-China, including six other existing global nighttime-light products, and Population (POP), Gross Domestic Products (GDP), built-up areas (BUA), as shown in Table 1.

**Table 1:** Data used to derive and validate PANDA-China with their sources.

Data	Description	Temporal range	Source
Population	The residential population in China.	1984-2020	China Statistical Yearbook 1984-2019
GDP	Gross domestic products in China.	1984-2020	China Statistical Yearbook 1984-2019
Built-up area	Annual built-up areas in China, including both urban and rural areas.	1978-2017	http://data.ess.tsinghua.edu.cn/urbanRuralChina.html
NTL data of Chen’s	Deep learning generated nighttime light dataset	2000-2020	https://doi.org/10.7910/DVN/YGIVCD
DMSP	Version 4 DMSP-OLS Nighttime Lights Time Series	1992-2012	https://eogdata.mines.edu/products/dmsp/#v4_dmsp_download
DMSP Extension	DMSP Nighttime Lights Extension	1992-2021	https://eogdata.mines.edu/wwwdata/dmsp/extension_series
DVNL	DMSP-like Nighttime Lights Derived from VNL (DVNL)	1992-2019	https://eogdata.mines.edu/wwwdata/viirs_products/dvnl/
NTL data of Li’s	A harmonized nighttime light dataset.	1992-2018	10.6084/m9.figshare.9828827.v2
NTL data of Zhang’s	DMSP-OLS data calibrated by ridged-line regression.	1992-2012	https://urbanization.yale.edu/data

### Implementation tasks

Two targets for PANDA-China are longer-period and higher consistency. The first part, aiming at target one, is to demonstrate NTLSTM routes and illustrates its process and components. The second part, aiming at target two, is to adapt RLOWESS to correct the temporal consistency of PANDA-China and systematically describes the assessments of NTLSTM and PANDA-China.

**Fig. 1:**
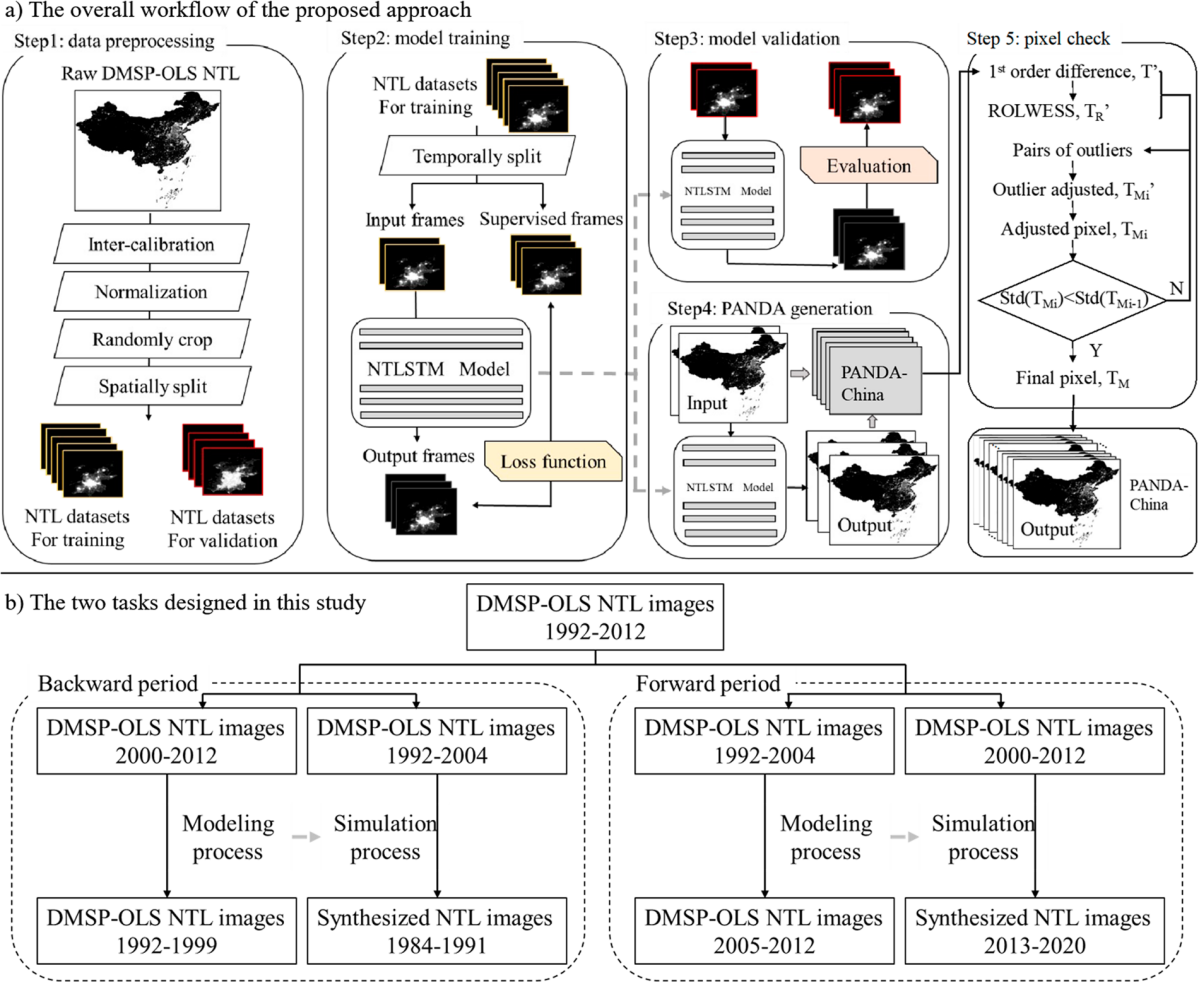
The proposed stepwise method. (**a**) The overall workflow of the proposed approach. (**b**) The two tasks designed in this study.

As illustrated in Fig. 1(a), we develop a stepwise method to achieve the extended NTL datasets consisting of the following five steps:

- Step 1: The raw DMSP NTL data is preprocessed by inter-calibration using methods proposed by^41^, followed by normalization. Then NTL training datasets and validation datasets are generated by randomly cropping and spatially splitting.
- Step 2: A nighttime light convolutional long short-term memory network (NTLSTM) is developed to model the inherent mechanism of dynamic changes of NTL datasets.
- Step 3: We utilize several assessment criteria for evaluating the performance of the proposed model in validation datasets.
- Step 4: The simulated NTL images of China (1984-2020) are generated using our properly trained NTLSTM.
- Step 5: The generated NTL data is temporally adjusted into a more consistent version of PANDA-China using MODEST (an adapted RLOWESS method).

We design two tasks in the training period (Fig. 1(b)). One is to backtrack the NTL data of the year 1984-1991, and the other is to forecast the NTL data of the year 2013-2020. The year 1984 is chosen as the end point of the backtracking task because Landsat-5, one of the most commonly used remote sensing imageries for early-year research, is mostly considered usable since circa 1984. The year 2020 is chosen as the end point of the forecasting task for the availability of other NTL datasets. The NTL data of 1992-2012 is split into two periods, 13 years of data for input and 8 years of data for supervision. Specifically, for the backtracking task, the deep learning network is supposed to be capable of utilizing the NTL data of the year 2000-2012 as input and backtracking the NTL data of the year 1992-1999. On the contrary, in the forecasting task, the deep learning network is designed to use the NTL data of the year 1992-2004 as input and to forecast the NTL data of the year 2005-2012.

### Nighttime light convolutional long short-term memory network

We apply tensor ${{\bf{T}}}_{y}^{1}$ with shape *y* × *h* × *w* to represent the input NTL patch sequence and tensor ${{\bf{T}}}_{y+z}^{y+1}$ with shape *z* × *h* × *w* to represent the target NTL patch sequence, where *y* denotes the length of inputted years, *z* represents the length of target year sequence, *h* *and* *w* denote the height and weight of each patch respectively:

1 $${{\bf{T}}}_{y}^{1}=[{{\bf{I}}}_{1},\,{{\bf{I}}}_{2},{{\bf{I}}}_{3},\,\ldots ,\,{{\bf{I}}}_{y}]$$

The **I**_*n*_ represents the NTL image patch at the *n*-th year, which is a *h* × *w* tensor:

2 $${{\bf{I}}}_{n}=\left[\begin{array}{ccc}{i}_{n}^{11} & \cdots  & {i}_{n}^{1w}\\ \vdots  & \ddots  & \vdots \\ {i}_{n}^{h1} & \cdots  & {i}_{n}^{hw}\end{array}\right]$$

**Fig. 2:**
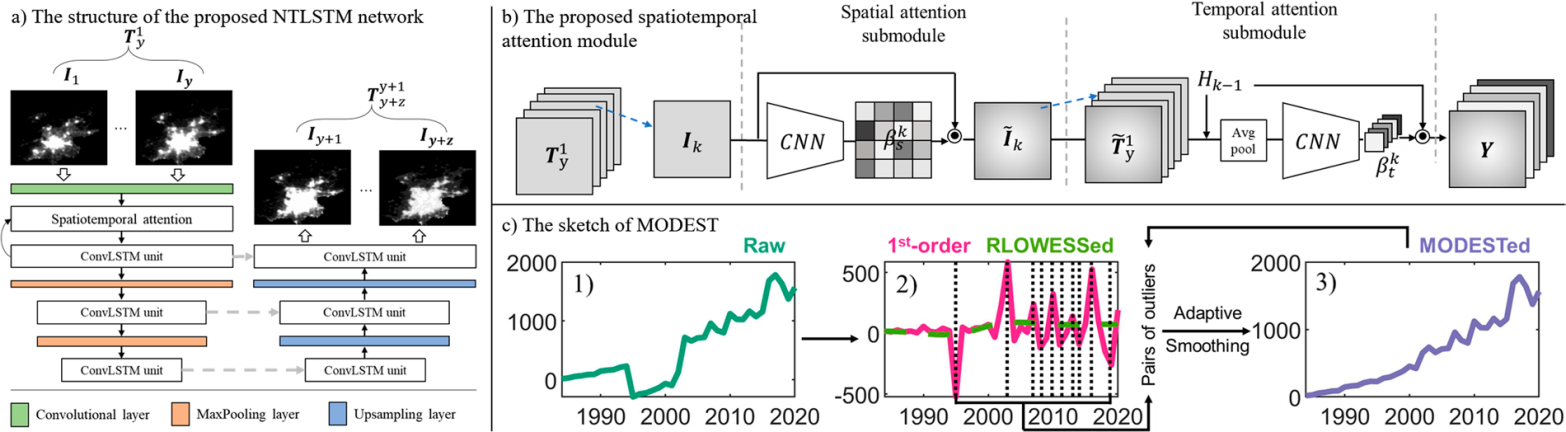
The overall methodology. (**a**) The structure of the proposed NTLSTM network. (**b**) The proposed spatiotemporal attention module. (**c**) The sketch of MODEST, of which 1) shows randomly generated time series, 2) shows the first-order difference of raw time-series (in magenta) and the RLOWESSed results (in green), and 3) cumulative sum time-series when replacing abrupt changing point value with RLOWESSed values.

Our ultimate goal is to learn a mapping function **F**(•) that can forecast the corresponding NTL sequence ${{\bf{T}}}_{y+z}^{y}$ via taking full use of the inputted ${{\bf{T}}}_{y}^{1}$. As illustrated in Fig. 2(a), we propose a nighttime light convolutional long short-term memory network (NTLSTM), which is regarded as our target mapping function **F**(•), consists of two main components: the spatiotemporal attention module and the convolutional LSTM unit. Other details of NTLSTM can be found in the supplementary material.

#### The spatiotemporal attention module

The attention module has been proposed to enhance the inherent feature representation capability of the networks and proved to be effective in quantities of previous studies^52–54^. Considering the information provided by the input NTL patches at different times and regions are unequally important for prediction performance, we propose a spatiotemporal attention module to implicitly learn spatiotemporal matrixes, which worked as weighting masks for further prediction. As illustrated in Fig. 2(2), the proposed spatiotemporal attention module consists of a spatial attention submodule and a temporal attention submodule, which automatically exploit different levels of importance of each NTL image patch sequence to generate spatiotemporally weighted feature maps **Y**.

The proposed spatial attention submodule is designed to adjust the input spatial features via calculating an attention matrix *β*_*s*_. This operation enhances or attenuates certain regions of the feature map based on their estimated attention weight. Here, we use two convolutional layers to learn the spatial attention matrix *β*_*s*_. Specifically, given the *k*^th^ patch feature, the spatially weighted feature ${\widetilde{{\bf{I}}}}_{k}$ is computed as a weighted summation using **I**_*k*_ and attention matrix ${\beta }_{s}^{k}$ as follows:

3 $${\widetilde{{\bf{I}}}}_{k}={\beta }_{s}^{k}{{\bf{I}}}_{k}$$

4 $${\beta }_{s}^{k}=\rho \left({\Phi }_{S1}{\Phi }_{S2}\left({{\bf{I}}}_{k}\right)\right)$$

where the *ρ* represents the softmax function, Φ_*S*1_ and Φ_*S*2_ are feed-forward neural networks with trainable parameters. Note that the learned spatial attention matrix *β*_*s*_ has the same height and width of the size of the input feature **I**_*k*_. While each input feature is attended over spatially via the spatial attention module, the temporal attention module is designed to calculate the temporal weight matrix *β*_*t*_ at each year. This temporal weight matrix *β*_*t*_ decides which year of the NTL patch sequence to pay attention to. Given spatially attended frames $\widetilde{{{\bf{T}}}_{y}^{1}}=[\widetilde{{{\bf{I}}}_{1}},\,\widetilde{{{\bf{I}}}_{2}},\widetilde{{{\bf{I}}}_{3}},\,\ldots ,\,\widetilde{{{\bf{I}}}_{y}}]$ and corresponding hidden state at (*k* − 1)^th^ of ConvLSTM *H*_*k*−1_, the temporal weight matrix at *k*-th ${\beta }_{t}^{k}$ is calculated as follows:

5 $${\beta }_{t}^{k}=\rho \left({\Phi }_{H}\left({H}_{k-1}\right)+{\Phi }_{I}\left(\widetilde{{{\bf{T}}}_{y}^{1}}\right)\right)$$

where the *ρ* represents a softmax function, Φ_*H*_ and Φ_*I*_ are feed-forward neural networks that are jointly trained with all other components of the proposed NTLSTM. Note that the temporal attention matrix *β*_*t*_ has the same length of input $\widetilde{{{\bf{T}}}_{y}^{1}}$.

#### The convolutional LSTM unit

The convolutional LSTM (ConvLSTM)^48^ captures spatiotemporal dependency in each NTL data sequence. Given the *k*^th^ spatiotemporally attended NTL patch features $\overline{{\widetilde{{\bf{I}}}}_{k}}$ in the inputted NTL feature maps **Y**, the input gate ${G}_{k}^{i}$, forget gate ${G}_{k}^{f}$ and output gate ${G}_{k}^{o}$ of the Convolutional LSTM (ConvLSTM) (please refer to the supplementary material for more details and illustrations) are calculated using following equations:

6 $${G}_{k}^{i}=\sigma \left({W}_{xi}\,\ast \,\overline{{\widetilde{{\bf{I}}}}_{k}}+{W}_{hi}\,\ast \,{H}_{k-1}+{b}_{i}\right)$$

7 $${G}_{k}^{f}=\sigma \left({W}_{xf}\,\ast \,\overline{{\widetilde{{\bf{I}}}}_{k}}+{W}_{hf}\,\ast \,{H}_{k-1}+{b}_{f}\right)$$

8 $${G}_{k}^{o}=\sigma \left({W}_{xo}\,\ast \,\overline{{\widetilde{{\bf{I}}}}_{k}}+{W}_{ho}\,\ast \,{H}_{k-1}+{b}_{o}\right)$$

9 $${C}_{k}={G}_{k}^{f}\,\bullet \,{C}_{k}+{G}_{k}^{i}\,\bullet \,\tanh \left({W}_{xc}\,\ast \,\overline{{\widetilde{{\bf{I}}}}_{k}}+{W}_{hc}\,\ast \,{H}_{k-1}+{b}_{c}\right)$$

10 $${H}_{k}={G}_{k}^{o}\,\bullet \,\tanh \left({C}_{k}\right)$$

where *σ* is the sigmoid function, (*) represents convolutional operator, and (•) is the Hadamard product. The *W* represents the weight matrix, each subscript has an obvious meaning. For example, *W*_*hi*_ is the hidden-input gate matrix, and *W*_*ho*_ is the input-output gate matrix, etc. The *b*_*i*_, *b*_*f*_, *b*_*o*_ and *b*_*c*_ are bias terms.

As shown in the above formulas, the ConvLSTM is a modification of LSTM, which replaces the fully-connected operators with convolutional operators. A ConvLSTM unit contains several ConvLSTM layers, each of which can extract the spatiotemporal features of certain frame $\overline{{\widetilde{{\bf{I}}}}_{k}}$. Thus, the ConvLSTM unit is capable to handle the inputted NTL sequence **Y**.

The proposed NTLSTM consists of two subnetwork structures: one is the encoding subnetwork *f*_*enc*_ (left part of Fig. 2(a)), and the other is the decoding subnetwork *f*_*dec*_ (right part of Fig. 2(a)), both of which are formed by stacking three ConvLSTM units. The initial states and cell outputs of the decoding subnetwork are copied from the last state of the encoding subnetwork. As shown in Fig. 2(a), the encoding subnetwork of NTLSTM extracts and compresses the spatiotemporal features from the input tensor ${{\bf{T}}}_{y}^{1}=[{{\bf{I}}}_{1},\,{{\bf{I}}}_{2},{{\bf{I}}}_{3},\,\ldots ,\,{{\bf{I}}}_{y}]$; the decoding subnetwork of NTLSTM unfolds the extracted features and predicts the final sequence ${{\bf{T}}}_{y+z}^{y+1}=[{{\bf{I}}}_{y+1},\,{{\bf{I}}}_{y+2},{{\bf{I}}}_{y+3},\,\ldots ,\,{{\bf{I}}}_{y+z}]$.

### Adjustment of outlier phases

Maximum-selection Of the Difference Enlarged by Smoothed Timeseries (MODEST) is proposed to adjust the inconsistency in marginal years between backward, original, and forward phases in raw PANDA-China. The challenge is to smooth the raw time-series while maintaining potentially helpful signals without ground truth. Therefore, MODEST is applied to PANDA-China pixel-by-pixel, followed by a spatially median smoothing window after its successful experiments on the basis of randomly generated time series data with a known shift up or down.

**Fig. 3:**
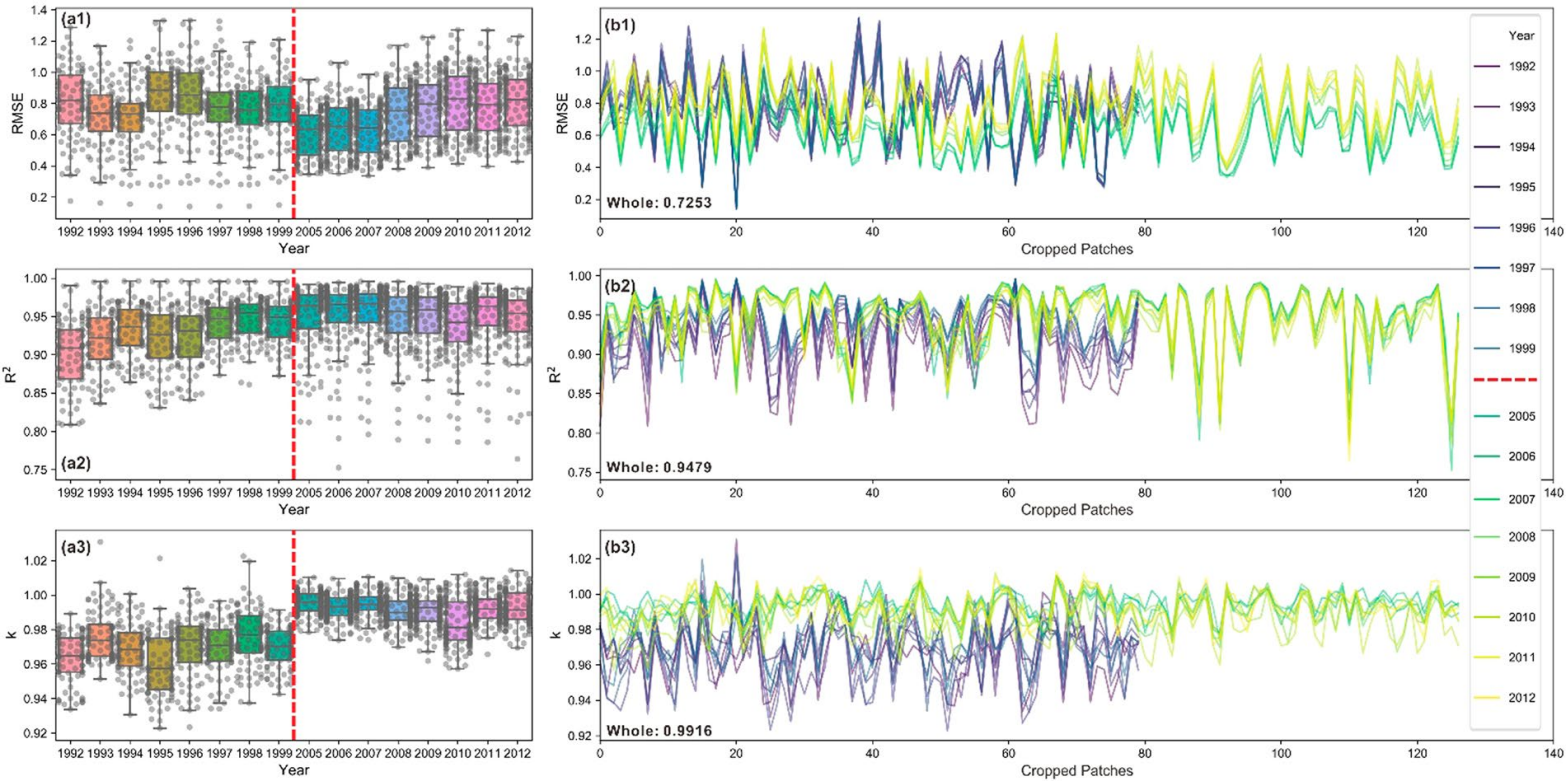
Temporal assessment of modeled results in randomly sampled cropped patches. The temporal trend of statistics of 79 and 126 cropped patches with all-zero ones excluded are shown: (**a1**) *RMSE*, (**a2**) *R*^2^, (**a3**) *k*; dots in each panel indicate data distribution. Statistic values from patch to patch: (**b1**) *RMSE*, (**b2**) *R*^2^, (**b3**) *k*, where colors indicate different years; note that the different numbers of cropped patches in backtracking and predicting model results from different patches with all zero values.

In general, MODEST includes two parts: detection and correction. To maintain potential valuable signals of raw PANDA-China (Fig. 2c-1), RLOWESS is applied to its first-order difference (in pink in Fig. 2c-2) instead of its raw time series and gets an RLOWESSed time series (in green dashed line in Fig. 2c-2). Values exceeding the three-standard deviation range are labeled as outliers (examples in black dashed line in Fig. 2c-2), where the highest and lowest values are detected as the start and end timestamp of the first outlier phase. It is then corrected by replacing values on detected start and end time with respective RLOWESSed values, and cumulatively summing the corrected first-order difference time series. This can be accepted as the final adjusted time series when the standard deviation of the corrected time series is lower than the results from the previous loop (Fig. 2c-3); otherwise, proceed to select the second pair of outliers and repeat the previous progress above. Further detailed processes and illustrations of MODEST and its results can be found in the supplementary information.

### Model assessment and product evaluation

Technical validation of PANDA-China mainly focuses on three parts: model assessment, product comparison with other existing datasets, and taking product correlation with socioeconomics as both the application and the assessment of PANDA-China.

We first assess the model based on the testing material, which can be divided into two phases: 1992-1999 from the backtracking task, and 2005-2012 from the predicting task. Widely adopted *Root Mean Square Error* (RMSE), linear regression determination coefficient *R*^2^ and its slope *k* are adopted to assess the accuracy, accordance, and over-/under-estimation, respectively. Multiple *RMSE*, *R*^2^, and *k* can be calculated between each pair of modeled and original patches, where temporal trend and annual uncertainties can be obtained and visualized. Secondly, our assessment focuses on differences between modeled and original summed values in the built-up areas (including both urban and rural areas). Temporally, 34 values are recorded in each year so that the accuracy of modeled results in previously and newly built-up areas can be measured annually. Spatially, differences in 1992-1999 and 2005-2012 in each China province are also used to evaluate the results of the model.

On the other hand, PANDA-China is compared with similar products by Li *et al*.^3^ and Zhang *et al*.^41^, on their spatiotemporal performance. Visual interpretation and correlation between NTL products and socioeconomic metrics are compared. More comparisons with other datasets can be found in the supplementary information.

Correlations between PANDA-China and socioeconomic variables are also calculated, as an assessment and an application. Since there is no ground truth for DMSP in 1984-1991 and 2013-2020, Pearson’s correlation R is calculated between PANDA-China and built-up areas (BUA), GDP, and population (POP) respectively, to build a consistent evaluation during the whole study period. For a better command of data performance, spatiotemporal evaluations are based on three manually selected phases: 1984-1991 (backtracked), 1992-2012 (modeled and original), and 2013-2020 (predicted).

## Data Records

PANDA-China is a prolonged artificial nighttime-light dataset of China ranging from 1984 to 2020, which has been produced using the developed Night-Time light convolutional Long Short-Term Memory network on the basis of DMSP-OLS. Model assessment shows the low error (RMSE: 0.73) and high accordance (R^2^: 0.95, linear slope: 0.99) at the pixel level, and well captures the temporal trends at newly-built urban areas while it slightly underestimates the intensity within older core urban areas. Pearson’s Rs are calculated between socioeconomic variables (BUA, GDP, POP) and PANDA-China in three phases, where reasonable values are presented and explained by history. PANDA-China provides consistent temporal trends, shows high accordance with socioeconomics, delineates road network, and thus is precious especially before 1992 and after 2013. PANDA-China helps to better demonstrate the dynamics of human activities in the long run and offers unprecedented opportunities to investigate economic or energy-related topics since 1984. PANDA-China is freely accessible at 10.11888/Socioeco.tpdc.271202^55^.

The data is stored in independent TIF format, each representing the night-time light data for a specific year, named *PANDA_China_Year.tif*. These files are organized in the following folder structure and are compatible with software such as ArcGIS. 

## Technical Validation

### Analysis of NTLSTM results

#### Temporal error distribution of modeled results

Temporally, the annual average *RMSE* of cropped patches reaches 0.47 with original data ranging from 0 to 63. By excluding the patches that contain only zero values, the average *RMSE* notably rises to 0.73. Inter-annual dynamics of *RMSE*, *R*^2^, and *k* with their uncertainty of backtracking and predicting models are visualized in boxplots in Fig. 3a1–a3 respectively, and the dots indicate the data distribution. These trends fluctuate with uncertainty in each period, but variances are still quite small. Data for each cropped patch are visualized in Fig. 3b1–b3. Note that different numbers of cropped patches here in predicting (126) and backtracking model (79) result from different numbers of cropped patches with all zero values.

#### Spatial error distribution of modeled results

**Fig. 4:**
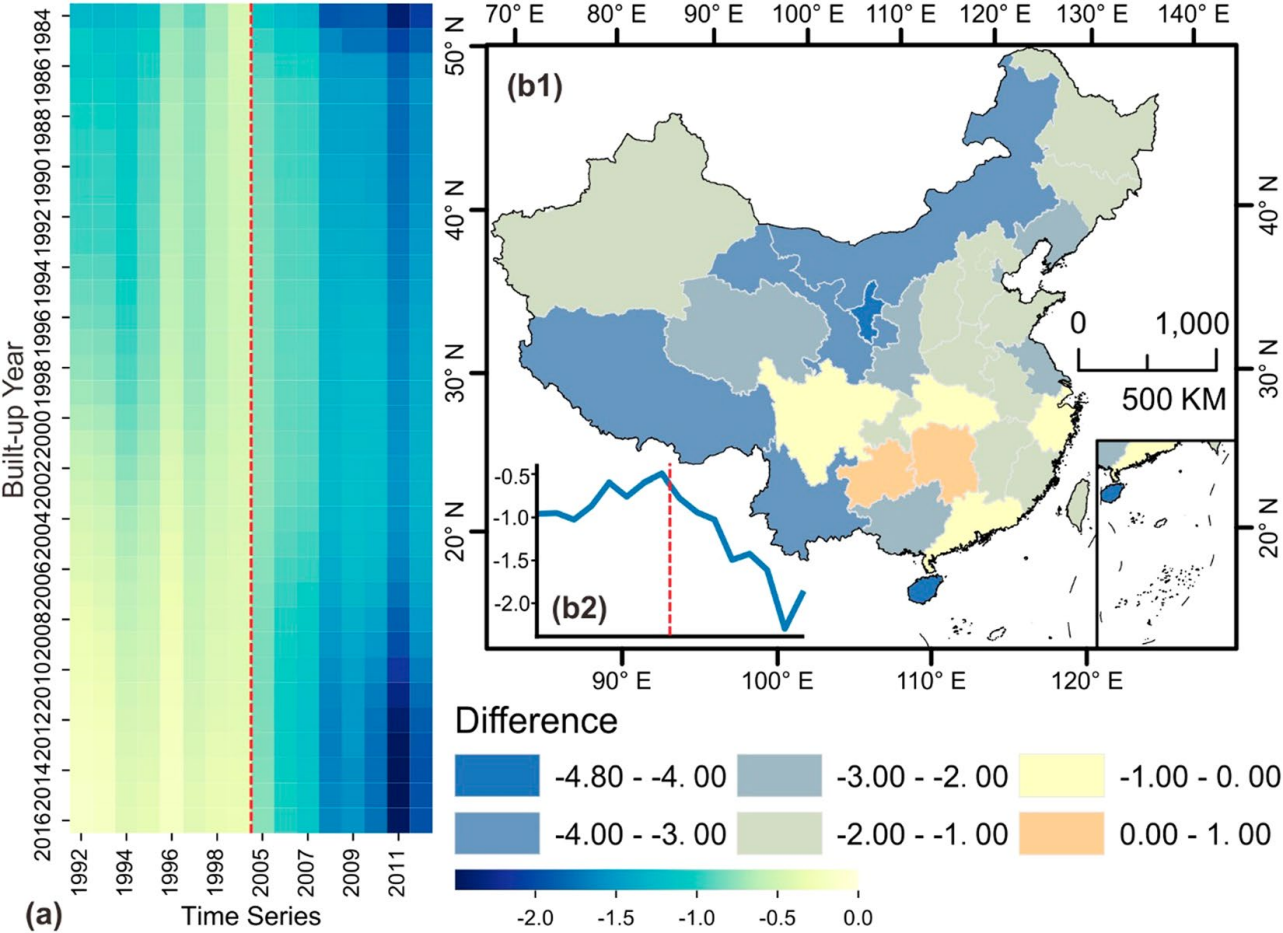
Spatiotemporal assessment of modeled results in urban areas. (**a**) Annual average of simple differences in areas with different built-up years. The upper one indicates older built-up areas and deeper blue indicates higher underestimation. (**b**) temporal and spatial heterogeneity of the averaged simple difference: (**b1**) spatial pattern of both underestimation and overestimation at the provincial level, and (**b2**) trend of averaged simple difference throughout the whole of China within built-up areas only.

To further test the model’s ability to depict night-time light variances, we investigate the built-up areas, known as the ad hoc areas of the NTL study. Temporally, the average of simple differences in all built-up areas throughout the whole of China is calculated annually, as shown in Fig. 4b2. Apparently, backtracking modeled results generally outperform predicting modeled results in highly developed areas, with the former within 0 to -1 and the latter exceeding -2 in 2011. Spatially, deeply investigating into areas with different built-up areas, we found older built-up areas are much underestimated (painted as deeper blue), while simple differences of newly-built areas or areas to be built keep closer to zero, as Fig. 4a shows. The results indicate that models are more powerful in describing the decreasing or increasing trend but limited in depicting variances in the established built-up areas. Apart from the underestimation in Fig. 4a, b2, there are still some slight overestimations when referring to spatial heterogeneity, such as that in Guizhou and Hunan provinces, as shown in Fig. 4b1. North-western China undergoes higher underestimation, while south-eastern China shows less underestimation.

### Comparison between PANDA-China and previous NTL datasets

#### Statistic comparison between seven datasets

**Fig. 5:**
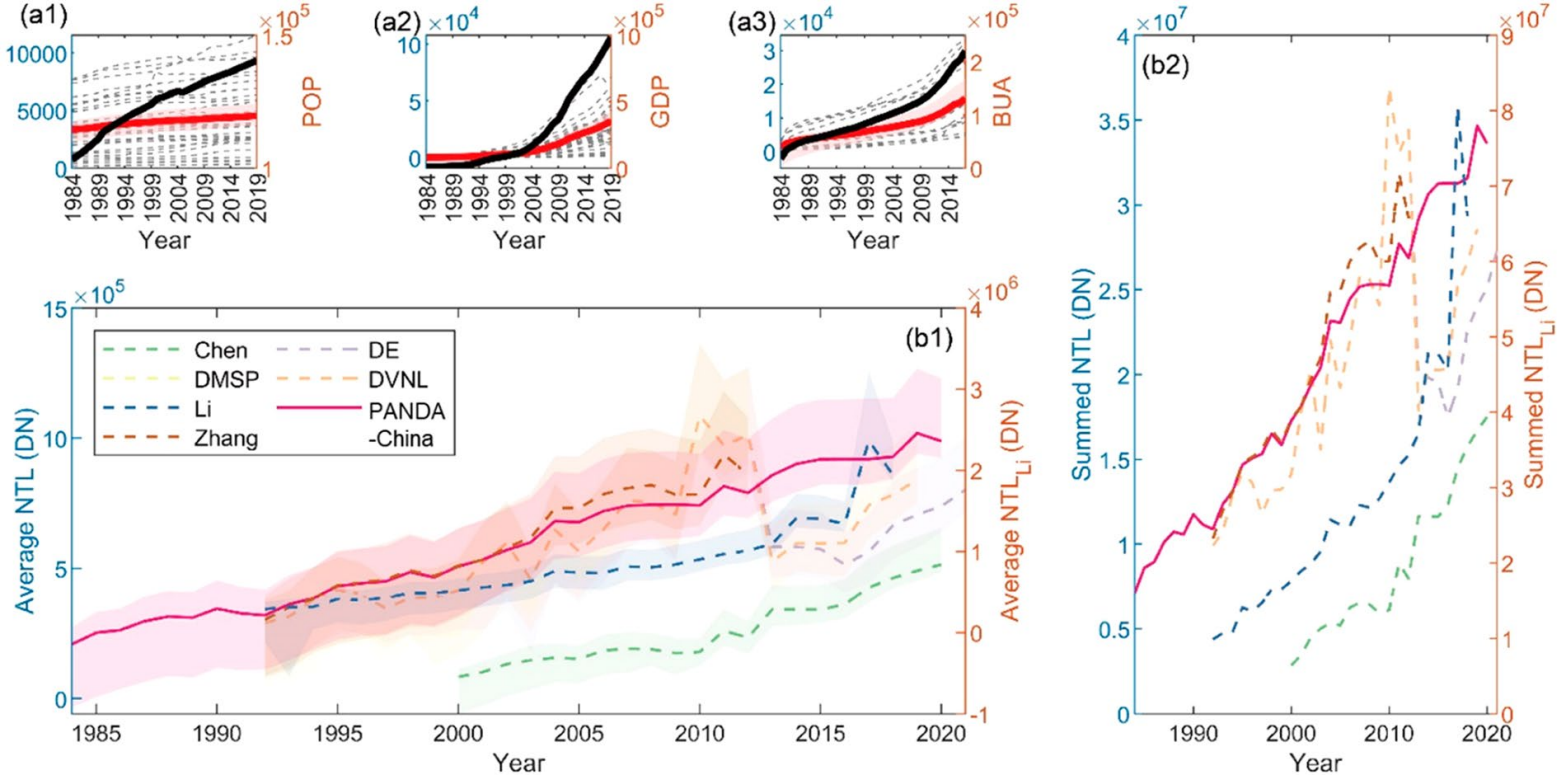
PANDA-China comparison with different socioeconomic variables and other datasets. Temporal dynamics of POP, GDP, and BUA have been shown in **a1**–**a3**, where black and red bold lines indicate their respective sum and average time-series, and grey dashed lines represent variables of each province. Seven NTL datasets have been summarized into province-wise average time-series (**b1**) and sum time-series (**b2**).

Direct comparisons are posted in Fig. 5. It provides two kinds of temporal dynamics of seven datasets, one is the averaged value of province-wise time-series with its temporal variance (Fig. 5b1), and the other is the summed value of the whole country (Fig. 5b2), both of which share the same figure legend. Besides, we also visualize the dynamics of POP, GDP, and BUA (Fig. 5a1–a3) for further indirect comparison. Black bold lines show the summed value of the whole country, and red bold ones show their respective averages, with pink areas indicating its temporal variance. Grey dashed lines in the background represent variable values in different provinces.

First, the most eye-catching advantage of PANDA-China is the time range prolonged by NTLSTM, compared to the other six sets of NTL datasets. It provides unique resources to understand social activities at night. Second, PANDA-China achieves high temporal consistency throughout its whole time-range both in average time-series and sum time-series, and its temporal trend cohorts with that of most datasets. Significant fluctuation mainly occurs around 2012 in other NTL datasets, like DE, DVNL, and Li’s, due to sensor degradation or the incorporation of cross-sensor information. Note that although the DN range of Chen’s seems much lower than DMSP-like datasets including PANDA-China in both average and sum time-series, its trend also shapes alike. Comparison of NTL temporal trends would be more helpful in comparing or applying NTL datasets since DN of DMSP-like NTL changes represents no explicit physical meaning (unitless).

To quantitatively compare NTL datasets against different socioeconomic variables, Pearson’s correlation analysis has been conducted on their same period, from 2000 to 2012, where seven datasets were further reduced to five types here since DE and DVNL share the same value with DMSP during this period. Major results have been summarized in Table 2. Two kinds of comparison are conducted. In the first type, the correlation between summed NTL and socioeconomic parameters (SOEC Param.) among all provinces has been calculated every year; and the results have been reported in Table 2 “By Year”. In the other type, the temporal correlation has been calculated in each province from various NTL sources, as listed in Table 2 “By Province” row. The average correlation of each and both types were calculated and reported as AVG shows.

**Table 2:** Correlation analysis between NTL products and socioeconomic parameters.

NTL Name	SOEC Param.	Chen	DMSP	Li	PANDA-China	Zhang
By Year	POP	0.5018	0.8403	0.6093	0.8692	0.7453
	GDP	0.8091	0.8788	0.7576	0.7510	0.8154
	BUA	0.8802	0.7535	0.8157	0.8093	0.8734
	**AVG**	**0.7303**	**0.8242**	**0.7275**	**0.8099**	**0.8114**
By Province	POP	0.5447	0.7889	0.7974	0.8866	0.6137
	GDP	0.8985	0.9085	0.5704	0.6083	0.8662
	BUA	0.8678	0.6062	0.8601	0.7960	0.7859
	**AVG**	**0.7703**	**0.7679**	**0.7426**	**0.7637**	**0.7553**
**AVG**		**0.7503**	**0.7960**	**0.7351**	**0.7868**	**0.7833**

Judging from the reported correlation, different kinds of NTL datasets emphasize various connections between night-time activities and socioeconomic development. From the profile’s view ("By Year” row), local night-time light mostly represents GDP and BUA in Chen’s and Li’s datasets, while it correlates well with all three parameters in DMSP, Zhang’s, and our PANDA-China datasets. From the perspective of time-series correlation ("By Province” row), it correlates higher with GDP and BUA than with POP in Chen’s and Zhang’s datasets, higher with POP and GDP than with BUA in DMSP, and higher with POP and BUA than with GDP in Li’s and our PANDA-China. Generally, an agreement would be reached on their correlation extent, no matter from the profile’s view or from time-series’ view; that is its stronger correlation with POP in the profile should persist in time-series. Chen’s, DMSP, and our PANDA-China meet this principle. PANDA-China also reaches the second-highest average correlation. More and thorough comparisons in detail can be found in the supplementary information.

Apart from the comparison of NTL values, that of spatial pattern should also be uncovered. Zhang’s and Li’s NTL datasets are selected as examples to be compared with the PANDA-China hereafter, since they share the same spatial resolution, and represent DMSP-based information and VIIRS-incorporated information, respectively.

#### Spatiotemporal comparison between representative datasets

Temporal consistent products derived from Li’s and Zhang’s methods are compared to PANDA-China in Shanghai- and Beijing-centred regions visually and throughout the whole China statistically. PANDA-China outperforms both of them in proper estimation and the description of road networks.

**Fig. 6:**
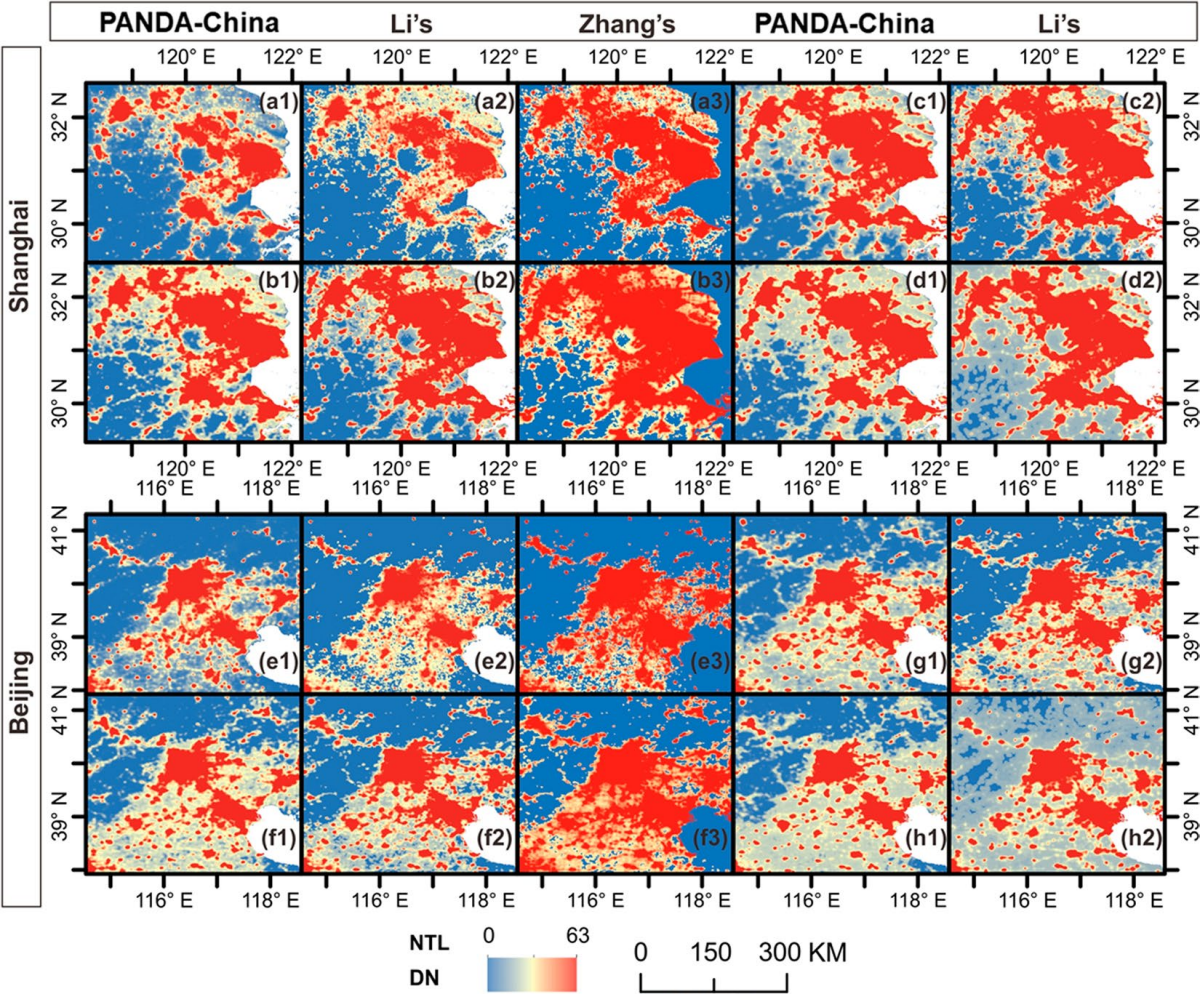
Comparison between PANDA-China and products from Li’s and Zhang’s methods (Li’s and Zhang’s hereafter) in Shanghai-centred and Beijing-centred regions. (**a1**–**a3**) and (**e1**–**e3**) show the spatial pattern of PANDA-China, Li’s and Zhang’s in 1992, the first year of published DMSP-OLS data; (**b1**–**b3**) and (**f1**–**f3**) show that in 2012, the last year of Zhang’s products. (**c1**–**c2**), (**g1**–**g2**) and (**d1**–**d2**), (**h1**--**h2**) compare PANDA-China and Li’s in 2013 and 2018, respectively.

In 1992, the earliest year of DMSP-OLS, our back-modeled result well captures the spatial pattern and the light intensity in both Shanghai and Beijing as Li’s (Fig. 6a1–a3,e1–e3). In contrast, slight overestimation exists at the fringe of urban areas resulting from the blooming in Zhang’s products. In 2012, which is a fast-developing period in both Shanghai and Beijing, PANDA-China delineates the road network much clearer than both Li’s and Zhang’s products. Specifically, the connection between Hangzhou and the city west to it is well reflected in the lower-left corner in Fig. 6b1, but not shown explicitly in Li’s and Zhang’s results, as shown in Fig. 6b2–b3. Similar situations can be seen in upper left corner of Fig. 6f1–f3.

Compared to adjustment of observations in Li’s and Zhang’s products, PANDA-China is not good at foreseeing light change. However, if we include such changes in the training process, such a capability to foresee light change can be maintained. As the lower-right corner of Fig. 6b1,c2 show, for the year of 2012, PANDA-China fails to predict the expressway between Shanghai and Hangzhou, while for the year of 2013, PANDA-China succeeds in bridging them. Similar phenomenon occurs in the west of Beijing (upper-left corner) in Fig. 6f1–g2. Surprisingly, Li’s product disconnects this road in Fig. 6g2 compared to Fig. 6g1. Besides, PANDA-China also shows a good capability to maintain the increasing spatial pattern, i.e. spatial consistency. As Fig. 6d1–d2,h1–h2 show, urban areas expanding from north-east to south-west in Shanghai-centred region, and from north to south in Beijing-centred region, inherit the pattern from Fig. 6c1–c2,d1–d2. In contrast, observations in Li’s product tend to underestimate the light intensity surrounding the urban areas, to overestimate at distant regions, and to obscure the road networks, as Fig. 6d1–d2,h1–h2 show. As Li’s product is considered to be better than Zhang’s in the literature^2^, we only perform the whole-China quantitative comparison between PANDA-China and Li’s product. As listed in Table 3, statistics of PANDA-China and Li’s product in these four years show close performance from 1992 to 2013, but PANDA-China outperforms Li’s product since then, which agrees with visual validation in Fig. 6. Please refer to the supplementary material for detailed annual statistics of the whole study period.

**Table 3:** Correlation between different NTL products and socioeconomic variables.

Products	Socioeconomic variables	1992	2012	2013	2018
PANDA-China	BUA	0.8576	0.8745	0.8703	0.8488*
	GDP	0.7833	0.7485	0.7322	0.5982
	POP	0.6378	0.7588	0.7446	0.7286
Li’s	BUA	0.8677	0.8657	0.8843	0.3997*
	GDP	0.7787	0.7343	0.7566	0.2809
	POP	0.6464	0.7213	0.7568	0.4632

* Correlation between products and BUA here is calculated in 2017 rather than 2018, owing to the data availability.

**Fig. 7:**
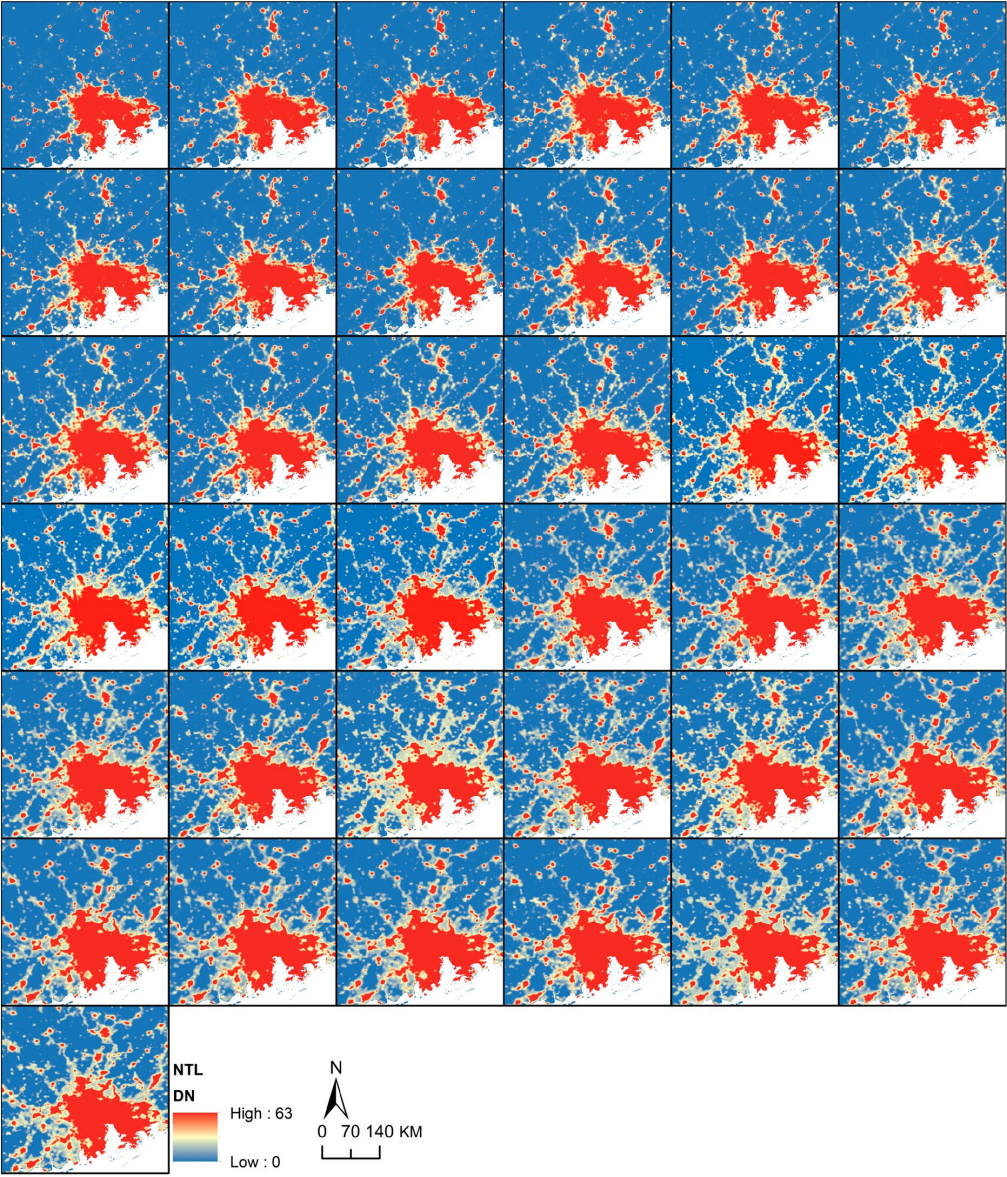
Spatial pattern of PANDA-China in the Guangzhou-centred region from 1984 to 2020.

With an unprecedented long time-span, PANDA-China helps to witness the connection between cities and villages during early years and project future expansion types. Taking the Guangzhou-centred region as an example, villages are nearly singly located around Guangzhou-centred urban agglomeration in 1984, and connections are gradually built between villages and between villages and cities during 1984 to 1992, as shown in the northern part of Fig. 7. Besides, PANDA-China manages to identify the major characteristics of the urban expansion. The urban area generally expands from urban fringe in Guangzhou during 1984 to 2020, but sometimes leaves a “hole” behind, mainly due to demographic reasons, such as mountainous or estuary regions, as shown in the southern part of Guangzhou from 2013 to 2020. Also, the comparison between PANDA-China with Landsat composites in this same area are shown (Figs. S7 ~ S8) in supplementary information.

### Spatiotemporal analysis of 37-year China nighttime light using PANDA-China

Three phases are selected accordingly: 1984-1991, 1992-2012, and 2013-2020, to excavate the controlling socioeconomic parameters of night-time light in different periods. Generally, PANDA-China is capable of characterizing the dynamics of BUA, GDP, and POP in each phase although their correlation varies in different provinces and phases.

**Fig. 8:**
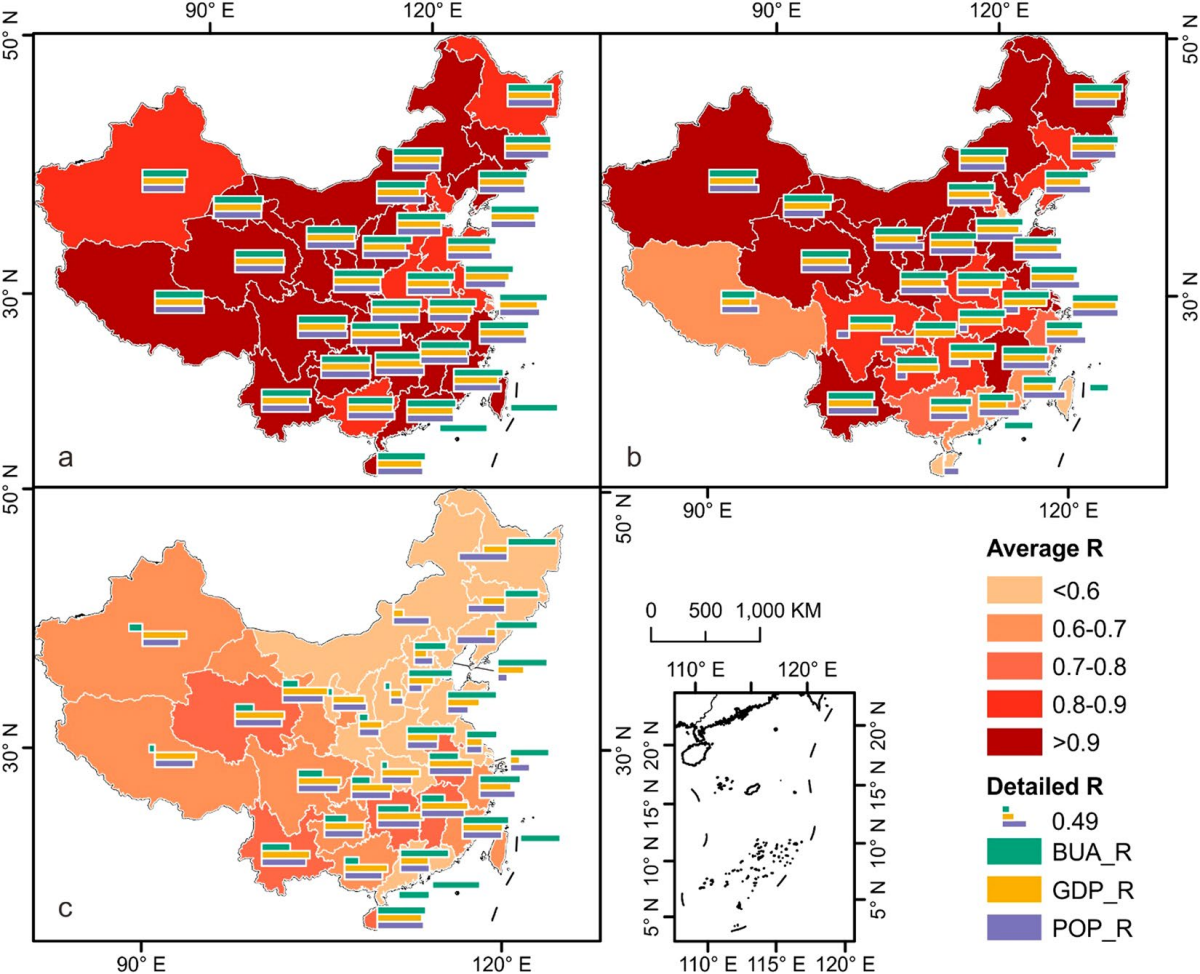
Spatiotemporal correlation between NTL and BUA, GDP, POP by provinces in each phase. 1984 to 2020 are divided into three phases according to model setting: (**a**) 1984-1991; (**b**) 1992-2012; (**c**) 2013-2020. The colors of provinces indicate the average value of three Pearson’s R.

In the first phase (1984-1991), NTL intensity is positively correlated with BUA, GDP, and POP to a large extent in all provinces (no GDP and POP statistical data available in Hongkong, Macau and Taiwan). This also indicates that urbanization, economic development and population increase all contribute to NTL in this phase throughout the whole China (Fig. 8a). While in the second phase (1992-2012), the situation changed. Generally, BUA and GDP still correlate well with NTL in all provinces except some in Hongkong, Macau, and Taiwan due to lack of statistic data. However, interaction between POP and NTL in each province varies largely. In Guizhou and Hunan provinces, their correlations reach a lower level (<0.5); and most notably, in Sichuan and Chongqing, there even exists negative correlation in the second phase (Fig. 8b). Most variances occur in the third phase. In this phase, NTL in each province results from different contributions. In south-eastern China, the relationships between BUA, GDP, POP, and NTL reach similar strong intensity as in first phase. In western China, NTL correlates well with GDP development and POP variances but shows lower relationship with BUA, especially in Xinjiang and Tibet. Not surprisingly, NTL correlates negatively to GDP and POP in north-eastern China, whose major contribution is BUA, as Fig. 8c shows.

### Supplementary information

Supplementary information for A Prolonged Artificial Nighttime-light Dataset of China (1984-2020)

## Data Availability

The programs used to generate all the results were Python 3.7, MATLAB (R2018b), and ArcGIS (10.4). The code and scripts used for training, testing, and predicting the NTL data are available in the open GitHub repository “https://github.com/xian1234/NTLSTM”, and the code for calibrating and validating the data is available at “https://www.mathworks.com/matlabcentral/fileexchange/119308-modest”.
