# Supplementary material for: A Prolonged Artificial Nighttime-light Dataset of China (1984-2020)
Source: Sci Data. 2024 Apr 22;11:414. doi: 10.1038/s41597-024-03223-1 (PMC11035565; doi:10.1038/s41597-024-03223-1)
Supplement: Supplementary file 1 — Supplementary information for A Prolonged Artificial Nighttime-light Dataset of China (1984-2020) [file 41597_2024_3223_MOESM1_ESM.docx]

Supplementary information for

A Prolonged Artificial Nighttime-light Dataset of China (1984-2020)

Lixian Zhang^1, 2, †^, Zhehao Ren^2, †^, Bin Chen^4, 5, 6^, Peng Gong^7, 8^, Bing Xu^2, 7, 9, *^, Haohuan Fu^2, 3, 7, 10, *^

1. High Performance Computing Department, National Supercomputing Center in Shenzhen, Shenzhen, China;

2. Department of Earth System Science, Tsinghua University, Beijing 100084, China;

3. Tsinghua University (Department of Earth System Science)-Xi'an Institute of Surveying and Mapping Joint Research Center for Next-Generation Smart Mapping, Beijing 100084, China;

4. Future Urbanity & Sustainable Environment (FUSE) Lab, Division of Landscape Architecture, Faculty of Architecture, The University of Hong Kong, Hong Kong Special Administrative Region;

5. Urban Systems Institute, The University of Hong Kong, Hong Kong Special Administrative Region;

6. HKU Musketeers Foundation Institute of Data Science, The University of Hong Kong, Hong Kong Special Administrative Region;

7. Ministry of Education Ecological Field Station for East Asian Migratory Birds, Beijing 100084, China;

8. Department of Geography, Department of Earth Sciences and Institute for Climate and Carbon Neutrality, The University of Hong Kong, Hong Kong, China;

9. International Research Center of Big Data for Sustainable Development Goals, Beijing 100094, China;

10. National Supercomputing Center in Wuxi, Wuxi, China.

† These authors contribute equally.

* Corresponding authors:

Bing Xu (bingxu@tsinghua.edu.cn), Haohuan Fu (haohuan@tsinghua.edu.cn)

**This file includes:**

Supplementary text:

NTLSTM details

MODEST algorithm

Comparison between pre- and post-MODEST products

Comparison between PANDA-China and daytime remote sensing data

Spatiotemporal variation analysis of 37-year China nighttime light using PANDA-China

Figures S1 to S8

Tables

**Supplementary text**

# 1. NTLSTM details: Normalization and loss function

As proved in several previous studies (Luo, Li et al. 2016, Mishkin, Sergievskiy et al. 2017), the input data size, which brings additional information and training samples for convolution filters, plays a distinct and important role in deep learning network. However, while accuracy depends largely on the size of the training image, the required computations and GPU memory grow quadratically with the data size. Therefore, as a compromise, enlarging the receptive field leads to reduced batch size, with the limitation of the GPU memory size and the network architecture. As proved by Wu and He (2018), batch normalization’s error increases rapidly when the batch size becomes smaller, caused by inaccurate batch statistics estimation. To solve the dilemma, we replace the batch normalization layer with a group normalization layer, which maintains a stable and better performance in small batch sizes compared with that of batch normalization. The group normalization layer $S_{i}$ is defined as:

$S_{i}=\left\{ k \right| k_{N}=i_{N},\left\lfloor\frac{k_{C}}{C/G} \right\rfloor=\left\lfloor\frac{i_{C}}{C/G} \right\rfloor\}$	(11)

where $G$ is the number of groups, which is a pre-defined hyper-parameter. $\left\lfloor\cdot\right\rfloor$ is the floor operation, $\left\lfloor\frac{k_{C}}{C/G} \right\rfloor=\left\lfloor\frac{i_{C}}{C/G} \right\rfloor$ means the indexes $i$ and $k$ are in the same group of channels along the $C$ axis.

Considering the NTL data contains a number of low NTL density regions, we apply the weighted mean square error (MSE) loss for training the NTLSTM model more efficiently. Hence, the ultimate goal can be transformed to build the mapping function $\boldsymbol{F}(\cdot)$ via our proposed NTLSTM to minimize the loss function:

$arg\min_{\boldsymbol{F}} {w\left\| \boldsymbol{I}- \tilde{\boldsymbol{I}} \right\|}_{2}$	(12)

where $\boldsymbol{I}$ and $\tilde{\boldsymbol{I}}$ are the output and ground-truth NTL images separately. The $w$ is another hyper-parameter and represents the loss weight, which is defined as:

$w_{i,j}=\left\{ \begin{aligned} 0.5, \tilde{\boldsymbol{I}_{i,j}}<0 \\ 1, elsewise \end{aligned} \right.$	(13)

We train the proposed NTLSTM with the Adam optimizer proposed by (Kingma and Ba 2014), the learning rate is initialized as 0.0001 with a reduced rate of 0.5 after every 5 epochs. We used random shuffling mini-batches for learning with a total of 30 training epochs. It took about 12h to train our proposed NTLSTM at an NVIDIA 2080Ti GPU.

# 2. MODEST algorithm

From time to time, time series data encounter individual outliers or even anomalous periods, which affect their further use. Anomalous time periods have new characteristics compared to individual outliers, for example, the prerequisite for correcting it is to determine the start and end points of this period, thus making it more challenging than dealing with individual outliers. Geography is still an important vehicle for studying population activities in the era of spatiotemporal big data, and remote sensing is gradually becoming an important tool for studying this topic. The problem of time series anomalous time periods is particularly serious in the analysis of time-series remote sensing image overlays. The common remote sensing primary products have the problem of insufficient time series continuity, such as MODIS-NDVI, DMSP_OLS, NPP_VIIRS/DNB, etc.

Even though time series outlier correction has a prominent place in spatiotemporal big data, no dedicated technical invention (software or tools) with a bottom threshold of use has been produced so far. While individual outlier correction already suffers from these problems, the correction of anomalous time periods faces a double lack of algorithms and technical inventions. Since systems with time series outlier correction are not available, users often make manual corrections without a uniform comparison method according to their own research scope and research needs, which raises the threshold for verifying repeatability and scalability and reduces credibility. If we can have the ability to correct them pixel-by-pixel at each time-point, it will fundamentally solve the problem of anomaly time correction even in time-series remote sensing image overlay.

There are many existing anomaly correction methods in the academic industry, such as moving average, moving median, Robust Locally Weighted Scatterplot Smoothing (RLOWESS), etc. after processing with the three standard deviation principle for screening. Unfortunately, these algorithms for correcting individual outliers cannot be used directly for correcting anomalous time periods.

In view of the fact that the above common algorithms cannot achieve the accurate detection of anomalous time period start points, and the correction of time-series oriented remote sensing images is even more impossible, a new technical invention is needed to solve this problem. The MODEST method (Maximum-selection of the Difference Enlarged by Smoothed Timeseries) is proposed to solve three technical problems: (1) accurately detecting the beginning and end points of anomalous timeseries; (2) preserving potentially valuable detail signals when correcting anomalous timeseries; (3) determining (3) determining the criteria for adaptive iterative correction of anomalous timeseries.

## 2.1 MODEST methods

### 2.1.1 Split adjustment into two targets

In order for the invention to have ubiquitous ability, it is necessary to assume that the time series contains multiple anomalous time periods to be corrected. Here, the accurate detection of the beginning and end points is split into two specific problems: How to detect the time series anomalies? How to filter from the outliers to get the beginning and end points of an anomalous time period?

The solution to the first question: The first-order difference series is first taken and then RLOWESS smoothing is performed on it, and three standard deviation ranges of the smoothed data are calculated. Using this range as a boundary, the abnormal high-value series and abnormal low-value series are determined.

The solution to the second question: According to multiple randomized trials, among a series of alternative anomalous values, the maximum anomalous high value and the maximum anomalous low value often correspond to the beginning and end of the most significant anomalous time period. In view of this, the alternative sequences of anomalous high values and alternative sequences of anomalous low values are sorted separately and sorted in descending order of absolute value for screening. The screening criteria are listed in the subsequent sections.

### 2.1.2 Adjustment scheme

The detection of the beginning and end points of anomalous time periods is the basis on which various options for correction are available. The difficulty of this problem is how to ensure the time series continuity while preserving the valuable fluctuation details. The present invention chooses the most direct and effective way:

(1) First, it distinguishes whether the anomalous time period belongs to the anomalous low-value class or the anomalous high-value class. When the maximum anomalous high-value time point is earlier than the maximum anomalous low-value time point, it shows an upward and then downward pattern and is identified as an anomalous high-value time period; on the contrary, it is an anomalous low-value time period.

(2) Second, only the beginning and end points of the anomalous time period are corrected, and the intermediate fluctuations are not smoothed in any way to retain detailed information.

In order to make the invention more generalizable, it needs to be made capable of handling timing data for more than one anomalous time period. Similarly, in order to ensure timing consistency while retaining potentially valuable detail signals, multiple anomalous time periods need to be corrected in a filtered manner. In other words, it is not possible to directly correct all alternative anomalies centrally at once, but iterative correction is required.

To make the present invention smarter, criteria need to be set for iterative correction and computed adaptively, reducing manual involvement. Several sets of iterative tests were made here, and it was found that in the adaptive calculation in real cases, when the overall standard deviation of the smoothed data was lower than that of the time series with anomalous time period data, the smoothing was sufficient to keep the balance principle (as in Fig.S1). Therefore, the overall standard deviation of the time series data is reduced as the judgment criterion to perform adaptive iterations.

Figure S1. Sketch for defining adaptive criteria in MODEST

## 2.2 MODEST pseudocode and application

### 2.2.1 MODEST pseudocode

The MODEST method based on the aforementioned operation has three significant advantages: (1) it can automatically correct the time series data containing any number of anomalous time periods; (2) while correcting the anomalous time periods, it retains the potentially valuable subtle fluctuation signals; (3) in addition to the conventional tabular time series data, the invention can correct the time series images with anomalous time periods by performing pixel-by-pixel time series processing. The application of the present invention on both time series data and time series images has been tested to have positive effects. Table S1 summarizes the pseudocode of the technical solutions of the present invention

Table S1. Pseudocode of MODEST

| Input: Original timeseries X | |
| --- | --- |
| Output: Adjusted timeseries X_adj_ | |
| 1 | Calculate first-order difference of X, and get Y; |
| 2 | Define frac% of Y to apply locally weighted regression, and name this neighbourhood as Y_k_； |
| 2.1 | In the first run, assign different weight W^(1)^ in different position within Y_k_, and take the weighted sum as a new value of Y_k_’, using weighted least square error calculation:  $\hat{Y}=X\cdot\left( X^{T}w^{(1)}X \right)^{-1}X^{T}w^{(1)}Y$ |
| 2.2 | In the second run, assign another different weight W^(2)^ in different position within Y_k_, where W^(2)^ can either be the same as W^(2)^ or not. Since this run, a penalty coefficient δ= W^(2)^(e/6s) has been multiplied to the weight, so as to lower the weight of the position with high mis-estimation, where least square error is adopted again. |
| 3 | Repeat these calculation until the target converges or exceeds the maximum running time, and denote it as Y’; |
| 4 | Calculate three standard deviation of Y’, and get the range as [Y_3std_down_, Y_3std_up_]; |
| 5 | Find all positions with Y value exceeds [Y_3std_down_, Y_3std_up_], sort them descendingly by its absolute value, and name these two new series as Y^up^(n), Y^down^(n). |
| 6 | On the basis of Y_(n)_, replace values to smooth the abrupt changes adaptivelyand get X_adj_. |
| 6.1 | In the first run, take Y^up^_(1)_ and Y^down^_(1)_as the start and end of the outlier phase, and replace the value at these two specific time with RLOWESSed ones, and denote its cumulative sum as X^(1)^_adj_; |
| 6.2 | Compare the standard deviation of X^(1)^_adj_ with the that of original timeseries. If smaller, stop loops; If not repeat calculations above and get X^(2)^_adj_. |
| 7 | Repeat step 6 until the adaptive criteria has been fulfilled, or the loop times exceed maximum, and then take X^(end)^_adj_ as the final X_adj_. |

### 2.2.2 MODEST application

A MATLAB-based application has been developed and is publicly available online for free according to MODEST. Its appearance is shown in Fig.S2, and the application itself can be acquired at https://www.mathworks.com/matlabcentral/fileexchange/119308-modest^[1]^.

Temporal inconsistency between phases may occur since NTLSTM is applied phase-by-phase. Fig.S8 visualizes the annual pair of pre- and post-adjusted nighttime light images of *Tai Hu*, a lake mainly in Jiangsu, China. Images show the raw PANDA-China spatiotemporal pattern, while colourful lines delineate adjusted pixels, where red boundaries imply elevated NTL values and blue ones imply the opposite.

Temporally, most changing points have mainly emerged since around 2004, which is the breakpoint of NTLSTM modeling. Spatially, most changing areas are located within the lake and its near boundaries. Generally, NTL intensity has been elevated from 2004 to 2012 within lake, and has been reduced since 2004 at lake boundaries. These phenomena implies that without auxiliary data, at dimmer region NTLSTM may overestimate NTL at already lighter regions while underestimate at dimmer regions, which is consist with our sensitivity analysis at section 5.2. However, although there may exists spatial or temporal inconsistency, the summed NTL values of this image suggests that the amount of NTL adjustment of the images is not comparable to its original intensity. PANDA-China is post-processed to be more applicable and reliable especially when used at small-scale spatial areas.

Figure S2. MODEST application appearance and its example in Taihu.

# 3. Comparison between products pre- and post- MODEST

## 3.1 Visual comparison with daytime and NPP-VIIRS data

In addition to being able to correct the time-series images at the data level to make them more consistent, the corrected time-series remote sensing data also have physical meaning and are quite realistic and valid. For example, in Fig.S3, the daytime optical image data shows that the site is an agricultural field, so it is reasonable to reduce the night light at the site after correction. Here we show the image directly corrected for time series with MODEST data, which has the problem of reduced spatial continuity. In the actual test, after the image is corrected with pixel-by-pixel time series, the corresponding changed pixel elements are also spatially filtered and smoothed to restore their spatial continuity; the parameters for spatial filter smoothing can also be set in the software.

Figure S3. Comparison of MODEST experimental results in an area of Songzi

In addition to comparison with optical images, higher spatial resolution NPP-VIIRS data can also be compared. For example, in Fig.S4, the corrected PANDA-China data are in better agreement with the finer NPP-VIIRS nighttime lighting data in a rural area of Beijing. There is an overestimation of the original PANDA-China, while the corrected PANDA-China after applying MODEST is able to be consistent with the NPP-VIIRS, adjusting the magnitude to a reasonable range.

Figure S4. Comparison of MODEST experimental results in an area of Beijing

## 3.2 Correlation analysis by province and by year

MODEST-corrected data can be compared not only by the naked eye, but also by highlighting the advantages after analysis with the temporal and spatial dimensions of socioeconomic indicators. As shown in Section 2.3.2, year-by-year multi-province PANDA-China can be analyzed with year-by-year multi-province Built-up Area (BUA), Population (POP) and GDP for spatial dimension, and also multi-year PANDA-China with multi-year values of the three socio-economic indicators for each province for temporal dimension The analysis can also be done at the temporal level by comparing the multi-year values of PANDA-China with the three socioeconomic indicators for each province.

Since DVNL and DE obviously do not have time series consistency, only Chen, DMSP, Li, PANDA, PANDAorg, PANDAnrb, and Zhang, the seven sets of data, are compared here, and the common time period of selected seven sets of data is 2000 to 2012 which is used in this comparison. The data PANDAorg and PANDAnrb are the results of the MODEST algorithm, and the difference is in the criteria of adaptive correction: the former criterion is that the standard deviation of the current correction curve is smaller than the standard deviation of the original PANDA, and the latter criterion is that it is smaller than the standard deviation of the series obtained in the previous iteration.

The spatial analysis level needs to be carried out year by year, with all provinces corresponding to PANDA-China and the three types of values for correlation analysis each year. When comparing PANDA, PANDAorg, and PANDAnrb, it is found that although MODEST is only a statistical sense algorithm, the PANDA corrected by MODEST still maintains a similar correlation with the original data as POP, GDP, and BUA, that is, the correction in a statistical sense does not destroy the physical meaning of PANDA itself, while in some years for example, in 2000, the corrected PANDAorg and PANDAnrb have higher correlations with POP and BUA than the original series. When comparing the PANDA series with other values, it is found that the Chen dataset maintains the best temporal consistency with the PANDA dataset in Figure 2 and 4, but the Chen dataset tends to have a lower correlation coefficient than PANDA when the time cross-section is analyzed with socioeconomic indicators, especially the value in 2000 is too low. And when the Chen, DMSP, Li, and Zhang data declined in correlation with socioeconomic indicators such as POP in 2009, around the Great Recession, PANDA still maintained better correlation. It reflects the strong ability of PANDA to express POP, GDP, and BUA after time enhancement.

At the level of time series analysis, PANDA-China and three socioeconomic indicators for 34 provincial units were analyzed separately for correlation, at which point the advantages of the MODEST algorithm were highlighted even more. In the comparison of all seven data sets, Chen, DMSP, and Zhang's data all have lower values for several provincial units, while both Li and PANDA data can better capture the correlation between the luminous time series and socioeconomic time series of each province, with Li being lower in Heilongjiang, Hubei, Hunan, and Jilin, and PANDA being lower in Tianjin and Taiwan. When comparing the three data sets within PANDA, it is clear that PANDAorg and PANDAnrb correlate better with socioeconomic indicators than PANDA.

Comparing both spatial and temporal dimensions, PANDAorg and PANDAnrb are superior to PANDA, which proves the advantage of MODEST algorithm from the data. However, in the MODEST algorithm, the two adaptive selection means do not show significant advantages and disadvantages in the analysis of this section, so given the practical experience, PANDAnrb is recommended to reduce the computational overhead.

Figure S5. Correlation between seven sets of luminous data and population, GDP, and built-up area by year

Figure S6. Correlation between seven sets of night-light data and population, GDP, and built-up area by province

# 4. Comparison between PANDA-China and daytime remote sensing data

Contours of PANDA-China from 1984 to 2020 are calculated and overlaid against Landsat composites using Google Earth. As Fig.S7 shows, peaks and pits indicated by contours in Guangdong Province denote city centers and forests, respectively, and move well along urban expansion.

|  |  |
| --- | --- |
|  |  |
|  |  |
|  |  |

Figure S7. Contours of PANDA-China ranging from 1984 to 1991 against Landsat composite using Google Earth

Also, similar depiction above are shown from 1984 to 2020 in Guangdong, China. Fig.11 shows the dynamics of PANDA-China dataset itself; in comparison, Fig.S8 shows how PANDA-China is conjured up with Landsat composites, a ubiquitously applied daytime remote sensing dataset. Combine two of them, and PANDA-China proves to be with high spatiotemporal consistency and good confidents.

# 5.  Spatiotemporal variation analysis of 37-year China nighttime light using PANDA-China

The night-time light was mainly located in key city agglomerations in 1984 (yellow patches in Figure S8. However, a bunch of light has progressed into much more regions in 2020 (mainly in green in Figure S8. This demonstrates that from the beginning of Reform and Opening-up tothe eradication of extreme poverty, light in China has progressed dramatically throughout the whole mainland China, regardless of various types of terrain, population, or culture. The reach-out of the light indicates the reach-out of infrastructure equity or modernity.

Figure S8. Shifts of night-time light centroid. Yellow patches in the map delineate the lit boundaries in 1984, while other colors represent the extent of light intensity in 2020. The weighted mean center was calculated annually thus shaping a curve in the zoomed-in figure, where the redder color shows a later year.

Hu's line, originally measuring the uneven population pattern, is widely taken as a geographic division line in China, whose formation derives from deeper controls like physical, social, or cultural factors [1]. The Centroid of night-time light was calculated as the centre position of all lit pixels in that year, taking the light intensity as weight. Judging from the comparative position of light centers and Hu's line, it's clear that not broken through as Hu's line was, the centroid was moving southward first and westward recently, approaching the line. This retrospectively affirms the West Development Strategy, and also prospectively advocates further devotion into western regions.

[1]	Hu, H. The distribution of china’s population - attached with statistical tables and density maps. Acta Geogr. Sinica 2, 33–74 (1935).

Figure S9. The mechanism of (a) cross-sensor calibration, and (b) our NTLSTM network.

Figure S10. The visualization of which patterns the NTLSTM has learned in the long-term NTL prediction. (a) the workflow of visualized pattern analysis, and (b) visualization results of the pattern learned by NTLSTM.

Figure S11. Contours of PANDA-China ranging from 1984 to 2020 against Landsat composite using Google Earth

Table S2. Correlation between different NTL products and socioeconomic variables (annual)

1. Zhehao REN (2023). MODEST (https://www.mathworks.com/matlabcentral/fileexchange/119308-modest), MATLAB Central File Exchange. Retrieved May 13, 2023.
